# The Use of Freestyle Libre Glucose Monitoring System and Diabetes Treatment Progression in Type 2 Diabetes Mellitus: A Retrospective Cohort Study in Saudi Arabia

**DOI:** 10.7759/cureus.79705

**Published:** 2025-02-26

**Authors:** Raed A Aljohani, Hanan N Altaib, Mostafa A Kofi

**Affiliations:** 1 Family Medicine, Prince Sultan Military Medical City, Riyadh, SAU; 2 Research Center, Prince Sultan Military Medical City, Riyadh, SAU

**Keywords:** continuous glucose monitoring (cgm), dibetes mellitus, freestyle libre, treatment intensification, treatment progression

## Abstract

Background

Type 2 diabetes mellitus (T2DM) is a prevalent chronic metabolic disease in Saudi Arabia, with a prevalence rate of approximately 25%. Traditional blood glucose monitoring methods, such as finger stick tests, provide limited insights into blood glucose measurements and fluctuations contributing to clinical inertia. The advent of continuous glucose monitoring (CGM) systems, such as the FreeStyle Libre glucose monitoring system, has transformed diabetes management by offering comprehensive exposure of glucose data to healthcare providers.

Objective

This study aims to evaluate the impact of the FreeStyle Libre glucose monitoring system on diabetes management intensification and treatment progression provided by healthcare specialists among T2DM patients in primary healthcare settings in Riyadh, Saudi Arabia.

Methods

An observational, retrospective, 24-week, two-arm study was conducted at Prince Sultan Military Medical City. The study involved 188 T2DM patients who were either using standard capillary glucose monitoring or transitioned to the FreeStyle Libre system, and it was based on clinical discretion rather than randomization. The primary outcomes were to evaluate the effect of FreeStyle Libre on treatment intensification provided by healthcare providers and changes in HbA1c levels. Data analysis included descriptive statistics and hypothesis testing using R software.

Results

Participants using the FreeStyle Libre glucose monitoring system experienced higher rates of medication intensification, and the use of insulin correction doses, and a significant reduction in median HbA1c levels was observed at three months (9.19% vs. 9.6%, p=0.047). However, at six months, the median HbA1c further reduced to 9.07%, though the difference between groups was not statistically significant. Despite these improvements, healthcare provider visits due to hyperglycemia were higher in the FreeStyle Libre group (p<0.001). There were no significant differences in hypoglycemia-related visits between the two groups (p=0.09).

Conclusion

The FreeStyle Libre glucose monitoring system was associated with increased treatment intensification and a significant reduction in HbA1c at three months compared to standard glucometers. However, by six months, the reduction in HbA1c was no longer statistically significant between groups. The increased healthcare provider visits in the FreeStyle Libre group may be attributed to heightened glucose monitoring awareness rather than the true worsening of hyperglycemia. While CGM offers advantages in diabetes management, its impact on long-term glycemic control remains uncertain. Further research is needed to confirm these findings, assess patient adherence, and evaluate the long-term effectiveness of continuous glucose monitoring in diabetes care.

## Introduction

Type 2 diabetes mellitus (T2DM) is a common chronic metabolic disease characterized by insulin deficiency and/or resistance and impaired blood glucose regulation [1]. In Saudi Arabia, the prevalence of T2DM is around 25% [2], making it a serious public health problem.

An accurate blood glucose measurement is important for effective diabetes management and blood sugar control [3]. Traditionally, blood glucose measurement in T2DM relied on intermittent finger stick testing, which provides limited information about glucose fluctuations during the day [3]. However, the development of continuous glucose monitoring (CGM) systems has revolutionized diabetes care [4].

FreeStyle Libre (FSL) uses subcutaneous, wired enzyme glucose-sensing technology to detect glucose levels in interstitial fluid, providing continuous valuable information on glucose trends [5]. The FSL system includes a small sensor applied on the skin to measure glucose levels in the interstitial fluid, eliminating the need for frequent finger stick checks [5]. This innovative technology has transformed diabetes self-management and enabled individuals to make timely adjustments to their treatment plans.

The FSL system has demonstrated several benefits in optimizing glycemic control and improving the quality of life for people with T2DM. Studies have shown that CGM leads to decreased HbA1c levels and reduced glycemic variability [6,7]. In addition, CGM is associated with a reduced risk of hypoglycemic events and increases the time in the range of blood glucose [4,8].

The use of the FSL system is effective for many different patient groups, including those receiving multiple daily insulin injections [9], pregnant women with diabetes [10], and the elderly with diabetes [11]. In addition to its impact on glycemic control, CGM has been associated with increased patient satisfaction and improved medication adherence [12].

Recent studies have also explored the cost-effectiveness of CGM. Long-term benefits in terms of reduced healthcare costs and improved outcomes have been reported [13,14]. In addition, CGM is useful in guiding treatment decisions, such as adjusting treatment regimens and optimizing insulin dosing strategies [15,16].

Despite the potential benefits, the use of CGM in T2DM management is not widespread. Factors such as cost, reimbursement issues, and patient perception may contribute to the underuse of this technology [17]. Globally, few observational studies have focused on treatment intensification with the use of FSL. A Canadian study illustrated that people with T2DM using FSL had a greater probability of treatment progression compared with blood glucose monitoring alone [18]. However, as evidence continues to accumulate on the effectiveness of CGM, it is essential to explore its implementation and impact on T2DM care in Saudi Arabia.

This study aims to investigate the use of the FSL system among patients with T2DM in Saudi Arabia. The study will evaluate the impact of CGM on diabetes management intensification using an FSL monitoring system in primary healthcare settings.

## Materials and methods

This study was performed according to the Declaration of Helsinki principles and was approved by Prince Sultan Military Medical City Scientific Research Center (IRB Approval No: E-2175).

Study design

This observational retrospective 24-week two-arm study was conducted in a single diabetes center at Prince Sultan Military Medical City, Riyadh, Saudi Arabia between August 2022 and March 2023.

Sample size

The sample size calculation was based on findings from a Canadian study that examined the impact of FSL on diabetes treatment progression. This study reported a relative risk (RR) of 1.86 to 2.81 (p < .001) for treatment intensification among FSL users compared to standard blood glucose monitoring [18]. Since absolute proportions were not stated, the required sample size was determined using the standard formula for comparing two proportions:

$$n = \frac{\left[ (Z_{\alpha/2} + Z_{\beta})^2 \times \left( P_1(1 - P_1) + P_2(1 - P_2) \right) \right]}{(P_1 - P_2)^2}$$

where Zα/2 = 1.96, Zβ = 0.84, and the reported effect size was used rather than assumed baseline rates. The calculation yielded a minimum required sample size of 71 participants per group to achieve 80% power at a 5% significance level. Our study included 94 participants per group, exceeding this threshold and ensuring adequate statistical power.

Participants

Adults with type 2 diabetes mellitus diagnosed for at least one year, using two or more insulin injections per day for at least six months, with HbA1c ≥9%, and attending the diabetes clinic in the primary health care center at Prince Sultan Military Medical City were included in the study. Individuals were excluded if they had type 1 diabetes, severe mental illness, pregnant women, or patients who were admitted to the hospital for any medical reason. Additionally, patients who were not willing to share glucose measurements or those who were not willing to use the FSL smartphone application were excluded. An informed consent was taken from the participants involved in the study.

**Figure 1:**
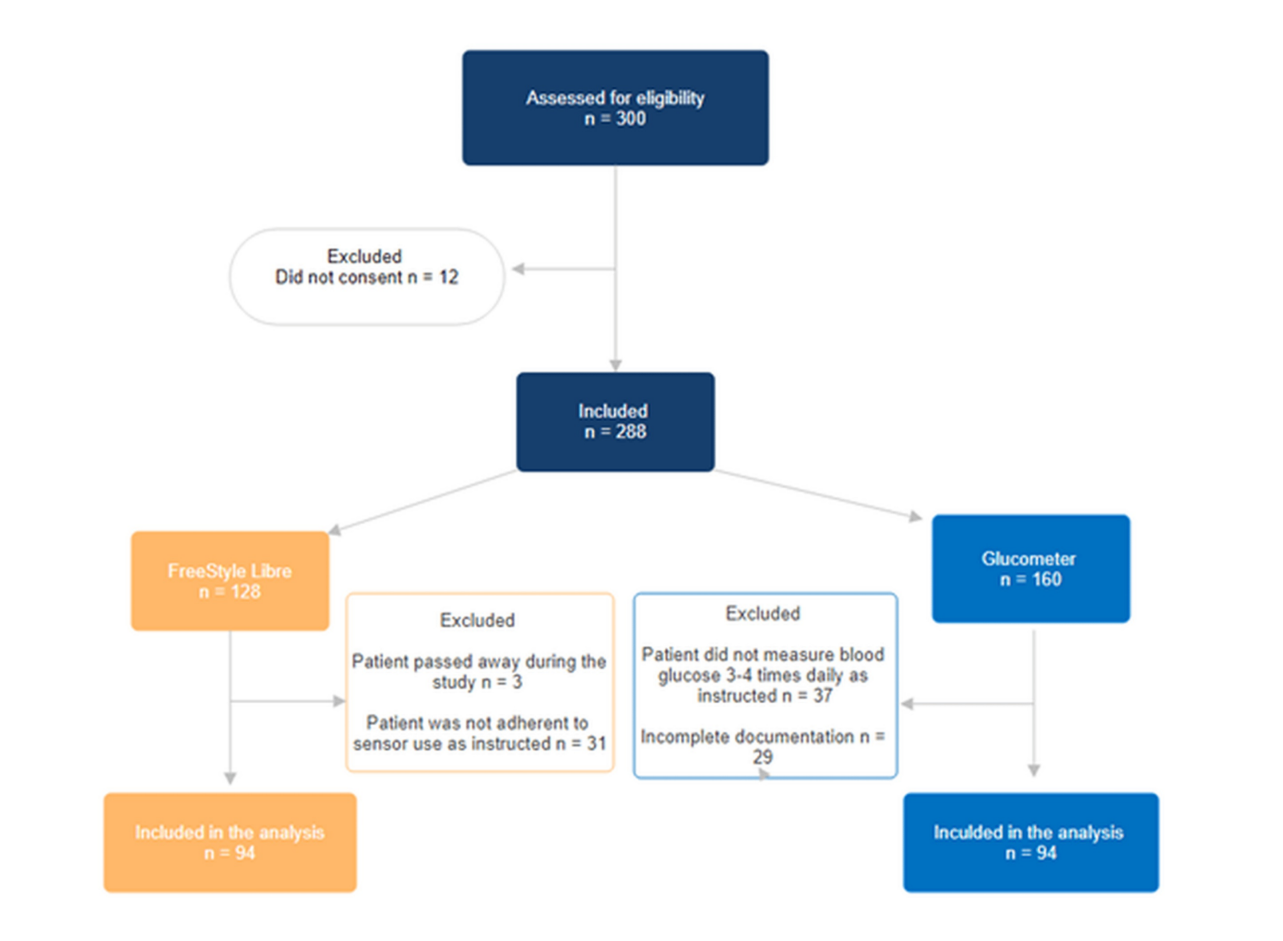
Patients Flowchart in the Study

The study involved two groups: participants using the FSL continuous glucose monitoring system and those using a glucometer. Out of 300 individuals assessed for eligibility, 12 were excluded for not providing consent, leaving 288 participants included in the study, of these, 128 were assigned to the FSL group and 160 to the glucometer group. In the FSL group, 34 participants were excluded due to death (n=3), and non-adherence to sensor use (n=31). Similarly, 66 participants in the glucometer group were excluded for not adhering to blood glucose monitoring instructions (n=37) or incomplete documentation (n=29). Ultimately, 94 participants from each group were included in the final analysis (Figure 1).

A total of 94 patients using standard capillary glucose monitoring were invited randomly to use FreeStyle Libre-2, a calibrated continuous interstitial glucose monitoring system (Abbott Diabetes Care, Witney, UK). A trained diabetes educator instructed the participants on the application of the sensors, which are worn on the back of the upper arm for 14 days to record subcutaneous interstitial glucose concentrations every minute.

The control group was made up of 94 patients using the standard capillary glucose monitoring, matched on demographic characteristics. These patients maintained their routine self-monitoring of blood glucose levels using their provided glucometer device to test their glucose levels at least four times per day, to record any symptoms of hypoglycemia, and to bring back their blood glucose log according to the follow-up visits.

All patients were invited to attend the diabetes treatment center for baseline, three-month, and six-month follow-up visits. Additionally, patients received follow-up telephone calls from diabetes educators every two to four weeks to titrate their insulin doses according to the measured blood glucose. Additional appointments were arranged for follow-up with the treating physician and educator.

To minimize selection bias, medical records were systematically reviewed, and consistent eligibility criteria were applied. Information bias was addressed by using standardized electronic health records, reducing the risk of recall errors. Residual confounding was mitigated through multivariate analysis, though unmeasured variables remain a limitation.

Variables

Patient demographic data (age, gender, diabetes duration, and background diabetes medications), type of glucometer device, frequency of blood glucose measuring per day, fasting blood glucose targets, postprandial blood glucose targets, HbA1c, hyperglycemia, and hypoglycemia events were collected from patient records. Data of anti-hyperglycemia medications that were added to participants’ plan of care by healthcare providers during the study period as well as insulin correction utilization were monitored for both groups. The primary outcome was the impact of using the FSL on diabetes treatment progression (Intensification) among patients with type 2 diabetes mellites and changes in HbA1c.

Data analysis

The data were extracted from an Excel sheet (Microsoft Corp., Redmond, WA, USA), cleaned, and imported into R software version 4.2.2 (R Foundation for Statistical Computing, Vienna, Austria). Descriptive statistics were used to calculate the median and interquartile range for the continuous variables and frequencies with percentages for categorical variables. Wilcoxon rank sum, Pearson's Chi-squared, and Fisher's exact tests were performed to identify factors associated with the use of FSL. The p-value of less than 0.05 was set as the significance level of the study.

## Results

The study included 188 patients with diabetes. The median age was 60 (Interquartile: 53, 67). Most patients were females (62%) and had no caregiver (53%). Half of the patients (50%) had their glucose level monitored using an FSL system and glucometer, respectively (Table 1).

**Table 1:** Demographic Characteristics of Patients with Diabetes ^1^n (%); Median (IQR)

Characteristic	N = 188^1^
Age	60 (53, 67)
Gender	
Female	117 (62%)
Male	71 (38%)
Caregiver	
No	100 (53%)
Yes	88 (47%)
Intervention	
Glucometer	94 (50%)
FreeStyle Libre system	94 (50%)

The median duration of diabetes mellitus was 17 years (IQR: 10-23 years). Among the patients, 86% were on multiple daily insulin injections (MDI), 97% were taking oral medications, 1.1% were using basal insulin, 26% were on glucagon-like peptide-1 receptor agonists (GLP-1RA) and 8.0% were on premixed insulin. Medication adherence was high, with 93% adhering to their regimen. The initial median HbA1c level was 10.00% (IQR: 9.30-11.00%), decreasing to 9.3% in three months (IQR: 8.70-10.00%). At six months, the median HbA1c further reduced to 9.07% (IQR: 8.34-10.00%).

Glucose levels in the study were categorized to highlight clinically significant thresholds. This included hypoglycemia, defined as Level 1 (<70 mg/dL) and Level 2 (<54 mg/dL), and hyperglycemia, with levels >180 mg/dL classified as elevated and >250 mg/dL as severe as seen in Table 2. Regarding glucose levels, 15% experienced levels below 54 mg/dL, 26%, 76%, and 68% experienced levels below 70 mg/dL, above 180 mg/dL, and above 250 mg/dL, respectively. Healthcare provider visits due to hyperglycemia or hypoglycemia occurred in 11% and 4.8%, respectively.

**Table 2:** Classification of Blood Glucose Levels: Hypoglycemia and Hyperglycemia Ranges

Category	Glucose Level (mg/dL)
Hypoglycemia Level 1	< 70
Hypoglycemia Level 2	< 54
Hyperglycemia	> 180
Severe Hyperglycemia	> 250

Most patients adhering to their medication regimen had their glucose monitored using the FSL (p=0.044). Also, most patients with hypoglycemia awareness used the FSL system for monitoring (p<0.001). The initial median HbA1c level for the glucometer group was 10.00% (IQR: 9.17-11.00%), decreasing to 9.60% (IQR: 9.00-10.10%) at three months and further to 9.00% (IQR: 8.39-10.45%) at six months. For the FreeStyle Libre group, the initial median HbA1c level was 10.01% (IQR: 9.30-11.33%), decreasing to 9.19% (IQR: 8.21-9.88%) at three months. However, at six months, the median HbA1c was 9.30% (IQR: 8.38-9.90%), showing a slight increase compared to the three-month follow-up. Therefore, patients using a glucometer had a higher median HbA1c at three months compared to those using the FSL system (median=9.6 vs 9.19, p=0.047). Additionally, most patients with glucose levels below 54 mg/dL (p<0.001), below 70 mg/dL (p<0.001), and above 180 mg/dL (p<0.001) were monitored using a glucometer (Table 3).

**Table 3:** Effect of FreeStyle Libre System on Diabetes Intensification ^1^Median (IQR); n (%) ^2^Wilcoxon rank sum test; Pearson's Chi-squared test; Fisher's exact test HbA1c: Hemoglobin A1C; MDI: Multiple daily insulin injections; GLP: Glucagon-like peptide

	Intervention		
Characteristic	Glucometer, N = 94^1^	FreeStyle Libre system, N = 94^1^	p-value^2^
Age	61 (53, 67)	60 (53, 67)	0.94
Gender			0.3
Female	62 (66%)	55 (59%)	
Male	32 (34%)	39 (41%)	
Caregiver			0.14
No	55 (59%)	45 (48%)	
Yes	39 (41%)	49 (52%)	
Duration of diabetes (years)	17 (11, 21)	16 (10, 25)	0.95
Insulin MDI			0.8
No	13 (14%)	14 (15%)	
Yes	81 (86%)	80 (85%)	
Oral			0.059
No	0 (0%)	5 (5.3%)	
Yes	94 (100%)	89 (95%)	
Insulin basal			0.5
No	94 (100%)	92 (98%)	
Yes	0 (0%)	2 (2.1%)	
Insulin prandial (novomix)			0.060
No	83 (88%)	90 (96%)	
Yes	11 (12%)	4 (4.3%)	
GLP1			1.00
No	70 (74%)	70 (74%)	
Yes	24 (26%)	24 (26%)	
Medication Adherence			0.044
No	10 (11%)	3 (3.2%)	
Yes	84 (89%)	91 (97%)	
Hypoglycemia Unawareness			<0.001
No	33 (35%)	80 (85%)	
Yes	61 (65%)	14 (15%)	
Initial HbA1c	10.00 (9.17, 11.00)	10.01 (9.30, 11.33)	0.5
HbA1c at 3 months	9.60 (9.00, 10.10)	9.19 (8.21, 9.88)	0.047
HbA1C at 6 months	9.00 (8.39, 10.45)	9.30 (8.38, 9.90)	0.5
Blood Glucose below 54 mg/dL			<0.001
No	67 (71%)	92 (98%)	
Yes	27 (29%)	2 (2.1%)	
Blood Glucose below 70 mg/dL			<0.001
No	50 (53%)	89 (95%)	
Yes	44 (47%)	5 (5.3%)	
Blood Glucose above 180 mg/dL			<0.001
No	12 (13%)	34 (36%)	
Yes	82 (87%)	60 (64%)	
Blood Glucose above 250 mg/dL			0.06
No	36 (38%)	24 (26%)	
Yes	58 (62%)	70 (74%)	

**Figure 2:**
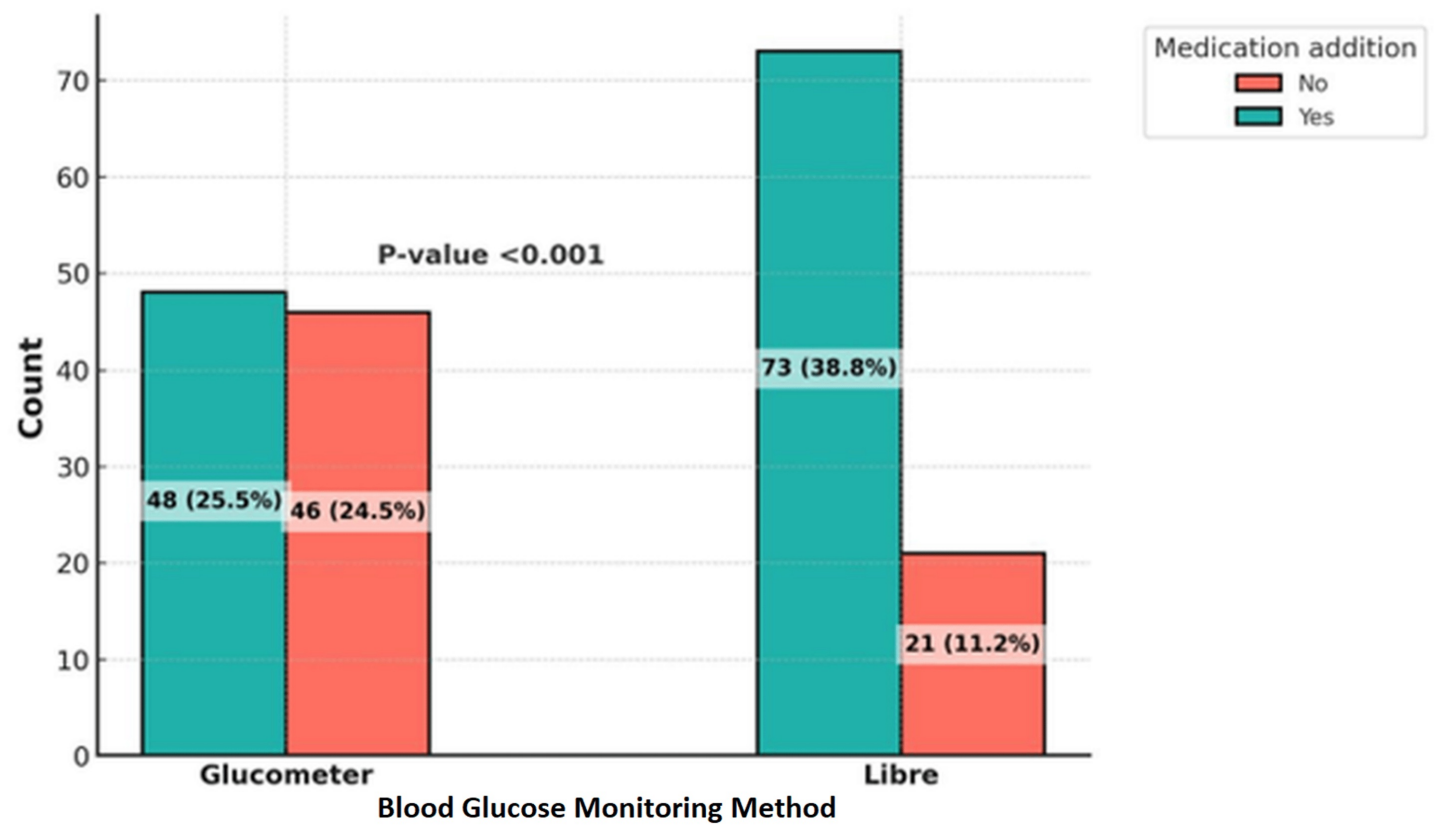
Impact of Blood Glucose Monitoring Methods on Medication Addition

The addition of medication was significantly more common in the FSL group compared to the glucometer group (p<0.001) (Figure 2). The treatment intensification rate was 25% (95% CI: 16.2%, 33.8%) in the glucometer group and 47% (95% CI: 36.9%, 57.1%) in the FSL group. Approximately 23% of patients used correction doses and 64% had medication additions. In the FSL group, metformin was added in 3.2% of the cases, empagliflozin was added in 40% of the patients, semaglutide was introduced in 22% of the cases, linagliptin was added for 19% of the patients, pioglitazone was added in 2.7% of the cases, and gliclazide was added in 1.6% of the patients. The use of insulin correction doses was significantly more common in the FSL group compared to the glucometer group (p=0.039). The addition of Semaglutide was significantly more often in the FSL group compared to the glucometer group (p<0.001). The addition of other drugs did not show a significant association (p>0.05) (Table 4).

**Table 4:** Impact of Blood Glucose Monitoring Methods on Medication Adjustment in Diabetes Management ^1^n (%) ^2^Pearson's Chi-squared test; Fisher's exact test

		Intervention		
Characteristic	Overall, N = 188^1^	Glucometer, N = 94^1^	FreeStyle Libre system, N = 94^1^	p-value^2^
The use of correction doses				0.039
No	144 (77%)	78 (83%)	66 (70%)	
Yes	44 (23%)	16 (17%)	28 (30%)	
Adding Metformin				0.2
No	182 (97%)	93 (99%)	89 (95%)	
Yes	6 (3.2%)	1 (1.1%)	5 (5.3%)	
Adding Empagliflozin				0.10
No	113 (60%)	62 (66%)	51 (54%)	
Yes	75 (40%)	32 (34%)	43 (46%)	
Adding Semaglutide				<0.001
No	147 (78%)	85 (90%)	62 (66%)	
Yes	41 (22%)	9 (9.6%)	32 (34%)	
Adding Linagliptin				0.3
No	152 (81%)	79 (84%)	73 (78%)	
Yes	36 (19%)	15 (16%)	21 (22%)	
Adding Pioglitazone				1.00
No	183 (97%)	91 (97%)	92 (98%)	
Yes	5 (2.7%)	3 (3.2%)	2 (2.1%)	
Adding Gliclazide				0.2
No	185 (98%)	94 (100%)	91 (97%)	
Yes	3 (1.6%)	0 (0%)	3 (3.2%)	

**Figure 3:**
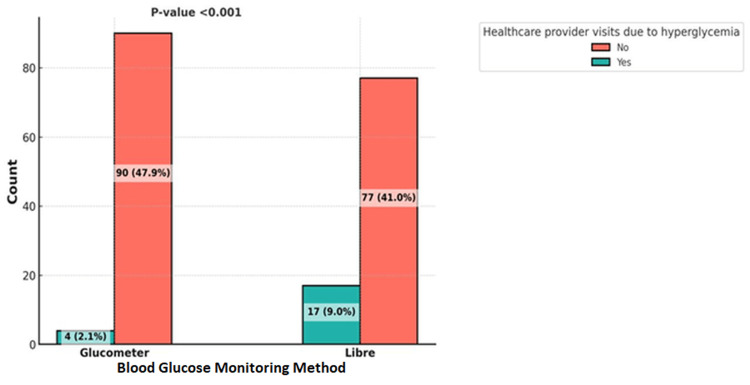
Comparison of Healthcare Provider Visiting of Patients Using Glucometer vs. FreeStyle Libre for Blood Glucose Monitoring (Hyperglycemia).

**Figure 4:**
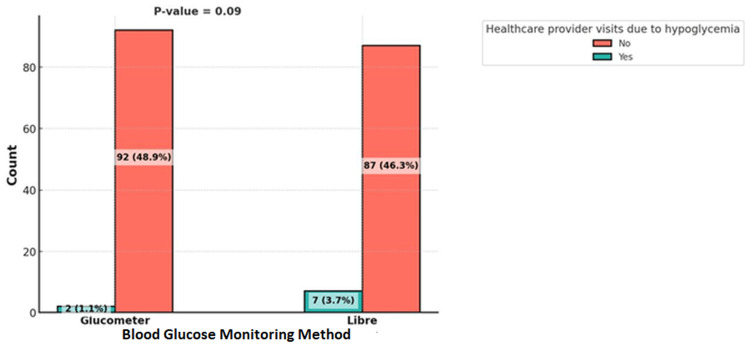
Comparison of Healthcare Provider Visiting of Patients Using Glucometer vs. FreeStyle Libre for Blood Glucose Monitoring (Hypoglycemia).

Healthcare provider visits due to hyperglycemia were significantly associated with the intervention used (p<0.001), with most of the patients who were visiting having their blood glucose monitored using the FSL system. However, visits due to hypoglycemia were not associated with the intervention used (p=0.09) (Figures 3, 4).

## Discussion

Continuous glucose monitoring (CGM) systems are increasingly used in diabetes management, helping optimize glucose control and achieve treatment targets in patients with type 2 diabetes mellitus (T2DM) [19,20]. This study assessed the impact of the FreeStyle Libre (FSL) system on diabetes treatment progression among patients with T2DM in a primary healthcare setting in Riyadh, Saudi Arabia.

Several studies have examined the efficacy of the FSL system in diabetes management. A clinical trial by Haak et al., conducted across three European countries, found no significant difference in HbA1c reduction between FSL users and those using self-monitoring of blood glucose at six months [7]. Similarly, our study showed no statistically significant difference in HbA1c levels between the FSL and glucometer groups at six months. However, at three months, we observed a significantly greater reduction in HbA1c among FSL users (p=0.047), aligning with Haak et al.'s findings that flash glucose monitoring led to short-term HbA1c improvements [7].

The initial improvement at three months, but lack of further reduction at six months, may be attributed to behavioral adaptation. Patients often show heightened adherence and motivation when initiating a new monitoring system, which may wane over time. Treatment fatigue, adaptation to CGM readings, and reactive rather than proactive therapy adjustments could contribute to diminished HbA1c benefits. Additionally, glycemic variability in CGM users may prompt frequent yet suboptimal dose adjustments, influencing long-term glycemic trends. Future studies should explore long-term adherence patterns and their role in sustained HbA1c improvements.

Despite more frequent treatment intensification in the FSL group, HbA1c levels at six months were slightly higher than at three months. This contradiction may result from differences in adherence patterns, potential overcorrections in response to real-time glucose fluctuations, or behavioral changes in response to increased glycemic awareness. Real-world patient behavior, provider decision-making, and socioeconomic factors may also have influenced these outcomes.

Significantly higher healthcare provider visits due to hyperglycemia were observed among FSL users. This may reflect increased glucose awareness leading to more frequent consultations rather than an actual increase in adverse hyperglycemia events. Without direct clinical confirmation, it remains unclear whether these visits were driven by clinically significant hyperglycemia or overreactions to real-time fluctuations. Future research should examine whether CGM use results in more frequent yet less critical healthcare interactions.

Previous studies have shown that standard glucometer users experience higher rates of hypoglycemia and hyperglycemia [21]. Galindo et al. found that FSL users had better glucose regulation, spending more time within the target glucose range and less time in hyperglycemia [22]. This suggests that while CGM improves glucose monitoring, it may also lead to increased patient-provider interactions as users seek reassurance or adjustments based on real-time readings.

Medication adjustments were more common in the FSL group (p<0.001), particularly with significantly higher use of Semaglutide and insulin correction doses (p=0.039). This indicates that CGM facilitates earlier recognition of glycemic trends, enabling proactive medication adjustments. Despite these benefits, the overall HbA1c reduction remained modest at both three and six months. Factors such as patient adherence challenges, variability in glucose readings, and differences in glycemic response among individuals could explain this modest improvement. Increased provider interactions might lead to frequent therapy changes but not necessarily better metabolic outcomes.

Our findings align with Harris and Levrat-Guillen, who reported that FSL use was associated with higher treatment intensification in patients with T2DM [18]. However, our study highlights increased healthcare provider visits among FSL users, suggesting that while CGM enhances treatment adjustments, it may also increase healthcare utilization driven by heightened awareness rather than true clinical necessity.

Several unmeasured confounders may have influenced our results, including baseline HbA1c, lifestyle factors, patient education, and socioeconomic status. Although selection bias was minimized through systematic record review and eligibility criteria, residual confounding remains a limitation. Future research should incorporate these factors to better assess the real-world effectiveness of CGM technology.

The FSL system is relatively new compared to traditional glucometers, and patient acceptance can be variable. However, our study found that patients with hypoglycemia awareness preferred the FSL system (p<0.001), indicating growing acceptance among diabetes patients in Saudi Arabia. Medication adherence was also significantly higher in FSL users (p=0.044), suggesting positive impacts on self-management behaviors. This aligns with findings from Hayek et al., who reported improved glucose management, dietary control, physical activity, and self-care behaviors with FSL use [23]. Previous studies have also demonstrated significant reductions in hypoglycemic episodes with FSL [24]. Although our study did not find a statistically significant difference in healthcare provider visits due to hypoglycemia between groups (p=0.09), prior research has shown that CGM users experience fewer severe hypoglycemic episodes. Differences in patient selection, real-world adherence, or the retrospective nature of our study may explain this discrepancy. Further research is needed to confirm whether long-term CGM use consistently reduces hypoglycemic events.

This is the first study evaluating the FSL system's impact on diabetes treatment progression in Riyadh, Saudi Arabia. Although the study had a strong response rate, limitations must be acknowledged. The retrospective design carries a risk of information bias, and as it was conducted in a single city, the findings' generalizability may be limited. Additionally, causality cannot be established, and unmeasured confounders might have influenced the results.

## Conclusions

The FreeStyle Libre system was associated with increased treatment intensification and a modest reduction in HbA1c at three months, though this effect diminished at six months. While CGM facilitates more frequent medication adjustments, it may also lead to increased healthcare provider visits driven by greater glucose awareness rather than worsening hyperglycemia. Future studies should explore the long-term impact of CGM on diabetes management, patient adherence, and clinical outcomes, while accounting for potential confounders such as socioeconomic factors and patient education.
